# Preparation, Mechanical Properties, and Degradation Behavior of Zn-1Fe-*x*Sr Alloys for Biomedical Applications

**DOI:** 10.3390/jfb15100289

**Published:** 2024-09-30

**Authors:** Wen Peng, Zehang Lu, Enyang Liu, Wenteng Wu, Sirong Yu, Jie Sun

**Affiliations:** 1 State Key Laboratory of Rolling and Automation, Northeastern University, Shenyang 110819, China; 2210240@stu.neu.edu.cn (W.W.); sunjie@ral.neu.edu.cn (J.S.); 2 School of Materials Science and Engineering, China University of Petroleum (East China), Qingdao 266580, China; luzehang163@163.com (Z.L.); enyangliu@126.com (E.L.); yusr@upc.edu.cn (S.Y.)

**Keywords:** Zn-1Fe-*x*Sr alloy, microstructure, grain refinement, mechanical properties, degradation behavior

## Abstract

As biodegradable materials, zinc (Zn) and zinc-based alloys have attracted wide attention owing to their great potential in biomedical applications. However, the poor strength of pure Zn and binary Zn alloys limits their wide application. In this work, a stir casting method was used to prepare the Zn-1Fe-*x*Sr (*x* = 0.5, 1, 1.5, 2 wt.%) ternary alloys, and the phase composition, microstructure, tensile properties, hardness, and degradation behavior were studied. The results indicated that the SrZn_13_ phase was generated in the Zn matrix when the Sr element was added, and the grain size of Zn-1Fe-*x*Sr alloy decreased with the increase in Sr content. The ultimate tensile strength (UTS) and Brinell hardness increased with the increase in Sr content. The UTS and hardness of Zn-1Fe-2Sr alloy were 141.65 MPa and 87.69 HBW, which were 55.7% and 58.4% higher than those of Zn-1Fe alloy, respectively. As the Sr content increased, the corrosion current density of Zn-1Fe-*x*Sr alloy increased, and the charge transfer resistance decreased significantly. Zn-1Fe-2Sr alloy had a degradation rate of 0.157 mg·cm^−2^·d^−1^, which was 118.1% higher than the degradation rate of Zn-1Fe alloy. Moreover, the degradation rate of Zn-1Fe-*x*Sr alloy decreased significantly with the increase in immersion time.

## 1. Introduction

Traditional biomedical metals, such as titanium alloys, stainless steels, and cobalt alloys, have been widely used in various clinical fields [1,2]. However, long-term implantation of these inert materials in the body may cause a series of adverse reactions. For example, these materials may release harmful metal ions or particles due to corrosion or wear, leading to inflammatory responses and tissue damage [3]. Therefore, most implanted metallic materials need to be surgically removed after fulfilling their clinical roles, which not only exacerbates the patient’s discomfort and expenses, but also increases the risk of postoperative infection [4,5]. Fortunately, the emergence of biodegradable implants has brought hope to change this situation. These implants can temporarily support the healing process of the diseased part of the human body, and then gradually degrade [6]. Biodegradable polymers are widely used in surgical sutures, cardiovascular stents, and orthopedic fixation materials [7]. Although they have been widely used in the medical field, the poor mechanical properties of most biodegradable polymers limit their clinical application [6]. In addition, the later developed polymer ceramic composites are only used in non-load-bearing parts because of their brittleness [8]. Recently, researchers have designed different kinds of biodegradable metallic materials [9,10,11,12]. Biodegradable metallic materials can naturally degrade in a physiological environment after completing clinical applications, and the degradation products can be absorbed and metabolized by the human body, and have reliable mechanical properties, effectively avoiding the aforementioned problems [13,14].

Recently, iron-based, magnesium-based, and zinc-based alloys have attracted great attention and have been extensively studied as biodegradable metallic materials [15,16,17,18]. Among these alloys, magnesium-based alloys have an excessively fast degradation rate, which may lead to excessive degradation and loss of function before clinical application is completed. Hydrogen gas will be released when the magnesium-based alloys are degraded, which inhibits the healing of biological tissues [19,20,21]. However, iron-based alloys have a slow degradation rate and often remain in the body for a particularly long time after completing clinical functions. Moreover, the degradation products of iron-based alloys are relatively stable and difficult to be absorbed by the human body. Long-term retention of these degradation products in the body may cause metabolic complications and syndromes [22,23]. Zinc is an essential trace element for the human body and is distributed in various tissues. More than 300 enzymes rely on zinc in the human body, so zinc is a nutritional element, which ensures the biological safety of zinc and zinc alloys as biodegradable metallic materials [24,25]. The standard electrode potential of zinc is higher than that of magnesium and lower than that of iron, and the corrosion rate of zinc in a neutral environment of the human body is more suitable for the interventional diagnosis and treatment fields, such as bone repair materials and stents [26]. The various advantages of zinc and zinc-based alloys make them the most promising degradable metallic materials for human implants [27].

However, the UTS and yield strength of pure zinc are poor and cannot meet the requirements of clinical applications [28]. In order to enhance the mechanical properties (such as UTS, hardness, etc.) of pure zinc, alloying treatment is usually carried out [29,30]. Iron is an essential trace element for the human body, and participates in various important metabolic activities, such as oxygen transportation and energy supply [31]. The mechanical properties of zinc can be improved significantly by the addition of a little amount of iron, without producing toxic effects on organisms. Zhang et al. [32] prepared Zn-0.5Cu-*x*Fe ternary alloy and found that FeZn_13_ phase was formed in the alloy, which could enhance the UTS of the alloy. Moreover, the addition of iron increased the degradation rate of Zn-0.5Cu-*x*Fe alloy. Strontium (Sr) is an essential trace element for the human body. Sr can promote the formation of osteoblasts, most of which are present in bones and teeth [33]. Li et al. [34] found that Sr played an irreplaceable role in bone mineral metabolism and was an ideal alloying element for designing biodegradable implant materials. In addition, the addition of Sr could reduce the grain size of alloys [35,36]. Therefore, adding a certain amount of Sr to Zn-Fe alloy is an effective approach to enhance its properties. Gutiérrez et al. [37] demonstrated that the hardness, yield strength, and the ultimate tensile strength of Zn-Al-Sr alloy were enhanced obviously by the addition of Sr, and the ductility did not decrease significantly.

In this research, Zn-1Fe-*x*Sr (*x* = 0.5, 1, 1.5, 2 wt.%) ternary alloys were prepared using a stir casting method, and the effect of Sr content on the phase composition, microstructure, tensile properties, hardness and degradation performance of Zn-1Fe-*x*Sr alloy was studied. The results could reveal the microstructure evolution mechanism and the reasons for changes in properties of Zn-1Fe-*x*Sr alloy, providing a foundation for the application of Zn alloys in the biomedical field.

## 2. Materials and Methods

### 2.1. Materials

**Figure 1:**
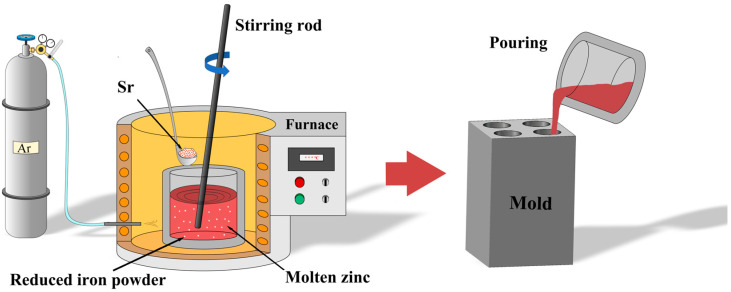
Preparation process of Zn-1Fe-*x*Sr alloy.

Pure zinc ingots (99.99 wt.%) were provided by Huludao Zinc Industry Co., Ltd., Huludao, China, and reduced iron powder (99 wt.%) was obtained from Sinopharm Chemical Reagent Co., Ltd., Shanghai, China. Pure Sr particles were purchased from Hebei Jiuyue New Materials Co., Ltd., Xingtai, China. The graphite crucible, stirring rod, and slag removal tool were preheated to 200 °C in a drying oven before use. A resistance furnace was used to prepare Zn-1Fe-*x*Sr (*x* = 0.5, 1, 1.5, 2 wt.%) ternary alloys. In order to avoid the oxidation of Zn melt, the argon gas was used as the protective gas and was injected into the resistance furnace. Zn ingots were added to the graphite crucible in the furnace, and the furnace temperature was set to 760 °C. After the Zn ingots were completely melted, the reduced iron powder was added to the melt and kept at 760 °C for 2 h. Then, the melt was cooled to 720 °C, and the pure Sr particles were added to the melt and kept at 720 °C for 40 min. In order to melt the iron powder and Sr particles as soon as possible and obtain a uniform distribution of Fe and Sr in the melt, the melt was stirred every 20 min for 3 min. Finally, the melt was cooled to 650 °C, and the slag was removed by a slag removal tool. Then, the melt was poured into a graphite mold which was preheated to 200 °C for cooling, and the Zn-1Fe-*x*Sr cast rods were obtained. The preparation process of Zn-1Fe-*x*Sr alloy is shown in Figure 1.

### 2.2. Microstructure Characterization

A wire-cutting machine was used to cut samples for microstructure characterization from the center of the cast rods. The size of the sample was φ25 mm × 15 mm, and the samples were first polished by 240#, 600#, 1000#, 1500#, and 2000# SiC sandpapers, and then polished on a polishing machine using 0.5 μm diamond polishing paste. Then, the samples were etched by 5 vol.% nitric acid ethanol solution and cleaned in deionized water, and finally cleaned in anhydrous ethanol and dried with cold air. The microstructure of the sample was characterized by a metallographic microscope (BX53M, Olympus, Tokyo, Japan), and the morphology of the sample after immersion was observed by a scanning electron microscope (JSM-7200F, JEOL, Tokyo, Japan). An energy-dispersive spectrometer (Aztec, Oxford, Britain) installed on a scanning electron microscope was used to analyze the composition of the alloy. An X-ray diffractometer (X’Pert PRO MPD, PANalytical, Almelo, The Netherlands) was used to identify the phase composition of the alloy. The Cu-Kα radiation was used and the scanning angle was 10°~75°, with a scanning rate of 2°/min.

### 2.3. Mechanical Property Test

A Brinell hardness test was conducted using a Brinell hardness tester (HB-3000B, Laizhou Weiyi Testing Instrument Manufacturing Co., Ltd., Yantai, China). A hard alloy ball with a diameter of 5 mm was pressed into the sample under a load of 2.452 kN, and after holding for 30 s, the load was removed. Then, the diameter of the indentation was measured and the hardness could be calculated. Five points on the sample were selected for testing, and the average value of these five points was used as the final hardness of the sample. Based on the ASTM-E8/E8M-11 standard, cylindrical tensile samples were cut from the mid-section of the cast rods using a computer numerical control (CNC) lathe. Tensile tests were conducted on an electronic universal testing machine (WDW-50E, Jinan Times Testing Machine Co., Ltd., Jinan, China) at room temperature, with a tensile rate of 1 mm/min.

### 2.4. Immersion Test

Based on the ASTM G31-2016 standard, the immersion tests were conducted in Hank’s balanced salt solution (HBSS). The HBSS was composed of 0.14 g/L CaCl_2_, 0.40 g/L KCl, 8.00 g/L NaCl, 0.09 g/L Na_2_HPO_4_·7H_2_O, 0.10 g/L MgSO_4_, 0.35 g/L NaHCO_3_, 0.06 g/L KH_2_PO_4_, and 1.00 g/L C_6_H_12_O_6_. The sample size for the immersion test was φ25 mm × 3 mm. Zn-1Fe-*x*Sr samples were immersed in HBSS at a temperature of 37 °C and were taken out every 7 days and weighed. The HBSS was replaced every 2 days in the immersion test. The degradation products should be removed before the samples were weighed. The samples after immersion were ultrasonically cleaned in 100 g/L NH_4_Cl solution at a temperature of 80 °C for 4 min, then cleaned with deionized water. Finally, the samples were dried with cold air, and the degradation products were removed. The degradation rate was calculated using the following equation:

(1) $$v=\frac{m_{2}-m_{1}}{S\cdot t}$$

where v is degradation rate, mg·cm^−2^·d^−1^; $m_{1}$ is the weight of Zn-1Fe-*x*Sr alloy, mg; $m_{2}$ is the weight of Zn-1Fe-*x*Sr alloy after immersion and the degradation products were removed, mg; S is the surface area of the sample, cm^2^; t is immersion time, d. In addition, in order to study the effect of Sr content on the long-term degradation behavior of Zn-1Fe-*x*Sr alloys, the alloys were immersed in HBSS for 28 days. After that, the samples were taken out and the degradation products were removed. Finally, the samples were weighed and the degradation rate was calculated.

### 2.5. Electrochemical Test

Electrochemical tests were performed in HBSS using a CS310H electrochemical workstation at a constant temperature of 37 °C. Zn-1Fe-*x*Sr alloys were encapsulated in epoxy resin, and a surface with an area of 1 cm^2^ was exposed. Then, the samples were polished and washed in anhydrous ethanol and dried. A three-electrode system was used, and Zn-1Fe-*x*Sr alloy was used as the working electrode. The platinum plate was used as the counter electrode, and the saturated calomel electrode (SCE) was used as the reference electrode. In order to make the open circuit potential (OCP) stable, the samples were immersed in HBSS for 60 min before the measurement. The potentiodynamic polarization curves were measured in the range of −0.5 to 0.5 V relative to the OCP, with a scanning rate of 0.333 mV/s. A Tafel extrapolation method was used to calculate the corrosion current density (*i_corr_*) and corrosion potential (*E_corr_*). The corrosion rate obtained by the electrochemical test was calculated using the following equation [38]:

(2) $$v_{c}=3.27\times 10^{-3}\;\frac{EW}{\rho }i_{corr}$$

where $\;v_{c}$ is corrosion rate, mm·year^−1^; EW is the equivalent weight, g/eq; *ρ* is the density of Zn, g/cm^3^; $i_{corr}$ is corrosion current density, μA/cm^2^. The electrochemical impedance spectroscopy (EIS) plots were measured in the frequency range from 10^5^ Hz to 10^−1^ Hz, using a perturbation amplitude of ±10 mV. Measurements were performed three times to ensure reproducibility of the results. The EIS data were fitted by ZSimpWin v3.60 software.

## 3. Results and Discussion

### 3.1. Microstructure

**Figure 2:**
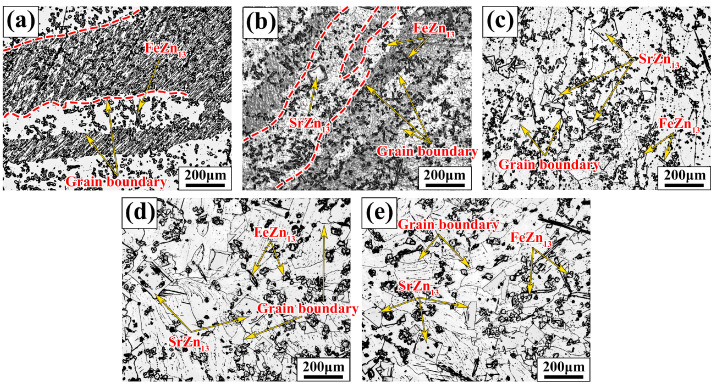
Microstructure of Zn-1Fe-*x*Sr alloys (**a**) Zn-1Fe; (**b**) Zn-1Fe-0.5Sr; (**c**) Zn-1Fe-1Sr; (**d**) Zn-1Fe-1.5Sr; (**e**) Zn-1Fe-2Sr.

**Figure 3:**
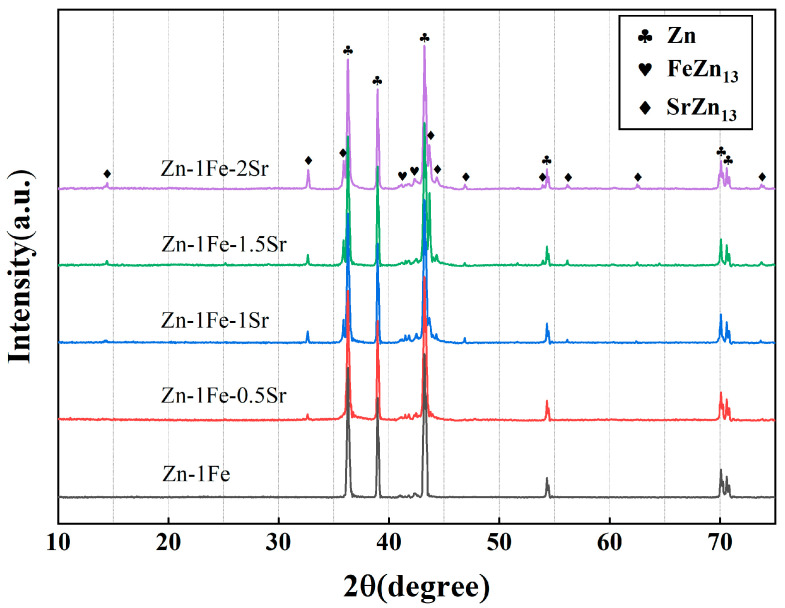
XRD patterns of Zn-1Fe-*x*Sr alloys.

Figure 2 shows the metallographic images of Zn-1Fe-*x*Sr alloys. As shown in Figure 2a, the grains in Zn-1Fe alloy were columnar and coarse, and FeZn_13_ particles were generated and distributed in the zinc grains. The irregular blocky SrZn_13_ phase was generated when the Sr element was added, as shown in Figure 2b–e. With the increase in the Sr content, the amount of SrZn_13_ phase increased. Furthermore, the grain size of η-Zn decreased with the increase in Sr content. Figure 3 shows the XRD patterns of Zn-1Fe-*x*Sr alloys. It can be seen that the Zn-1Fe alloy consisted of η-Zn and ζ-FeZn_13_ phase. When the Sr element was added, the diffraction peaks of SrZn_13_ phase were found in the XRD patterns. As the Sr content increased, the intensity of the diffraction peaks of SrZn_13_ phase increased, indicating an increase in the amount of SrZn_13_ phase.

**Figure 4:**
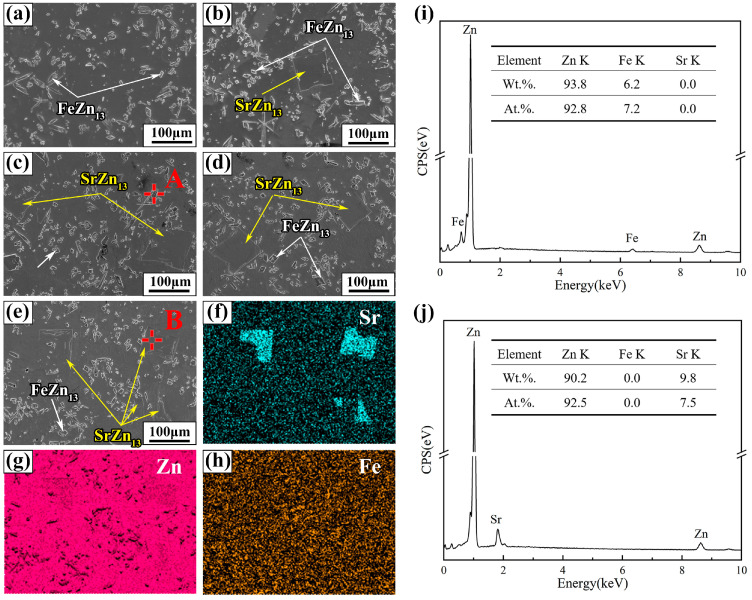
SEM images and EDS analysis of Zn-1Fe-*x*Sr alloys (**a**) Zn-1Fe; (**b**) Zn-1Fe-0.5Sr; (**c**) Zn-1Fe-1Sr; (**d**) Zn-1Fe-1.5Sr; (**e**) Zn-1Fe-2Sr; (**f**–**h**) EDS maps of Zn-1Fe-2Sr; (**i**) EDS analysis of point A; (**j**) EDS analysis of point B.

The SEM images and EDS analysis results of Zn-1Fe-*x*Sr alloys are shown in Figure 4. As shown in Figure 4a–e, two different second phases, granular and blocky, were found in the alloy matrix. The EDS mapping of Zn-1Fe-2Sr alloy indicated that Fe mainly existed in the granular second phase, while Sr mainly existed in the blocky second phase, as demonstrated in Figure 4f–h. The EDS quantitative analysis results of the two points marked as A and B are shown in Figure 4i,j. Point A was granular second phase composed of Zn and Fe, with a Zn/Fe atomic ratio of approximately 13:1. Point B was blocky second phase composed of Zn and Sr, with a Zn/Sr atomic ratio of approximately 13:1. Therefore, based on the EDS analysis results and previous XRD analysis, it can be determined that the granular second phase was FeZn_13_ and the blocky second phase was SrZn_13_.

As can be seen from the Zn-Fe binary phase diagram [39], Fe was nearly insoluble in Zn, and the Zn-1Fe alloy consisted of ζ-FeZn_13_ phase and η-Zn phase. During the cooling process of Zn-1Fe alloy, the δ-FeZn_10_ phase was generated in the Zn-Fe melt when the temperature dropped below the liquidus temperature. When the temperature of the melt dropped to 530 °C, a peritectic transformation between the δ phase and the melt occurred (L + δ→ζ), and the ζ phase was formed. As the temperature decreased, the ζ phase was formed continuously. When the temperature of the melt dropped to 419.5 °C, the eutectic reaction occurred (L→ζ + η), and the ζ-FeZn_13_ and η-Zn were formed. At the eutectic point, the content of Fe in the liquid phase was ~0.01 wt.% [39], so only a very small amount of ζ phase was generated in the eutectic reaction. The ζ phase continued to grow on the ζ particles formed previously. When the temperature decreased to the room temperature, there were only ζ-FeZn_13_ phase and η-Zn phase in Zn-1Fe alloy, and the ζ-FeZn_13_ particles were distributed in the η-Zn matrix. As can be seen from the Zn-Sr binary phase diagram [40], Sr was insoluble in zinc. During the cooling process of Zn-Sr alloy with Sr content less than 2 wt.%, the SrZn_13_ phase was formed in the Zn-Sr melt when the temperature dropped below the liquidus temperature. When the temperature of the melt dropped to 420 °C, the eutectic reaction (L→SrZn_13_ + η-Zn) occurred. As the content of Sr in the liquid phase was nearly 0% at the eutectic point, the amount of SrZn_13_ phase formed in the eutectic reaction was extremely small and could be ignored [40].

During the cooling process of Zn-1Fe-*x*Sr alloy, the FeZn_13_ and SrZn_13_ particles were formed and dispersed in the melt. These particles could serve as the heterogeneous nucleation sites for η-Zn grains, improving the nucleation rate. On the other hand, these particles could also restrict the growth rate of η-Zn grains, leading to the grain refinement of Zn-1Fe-*x*Sr alloy. Hence, the grain size of η-Zn decreased with the increase in Sr content, as indicated in Figure 2.

### 3.2. Mechanical Properties

#### 3.2.1. Brinell Hardness

**Figure 5:**
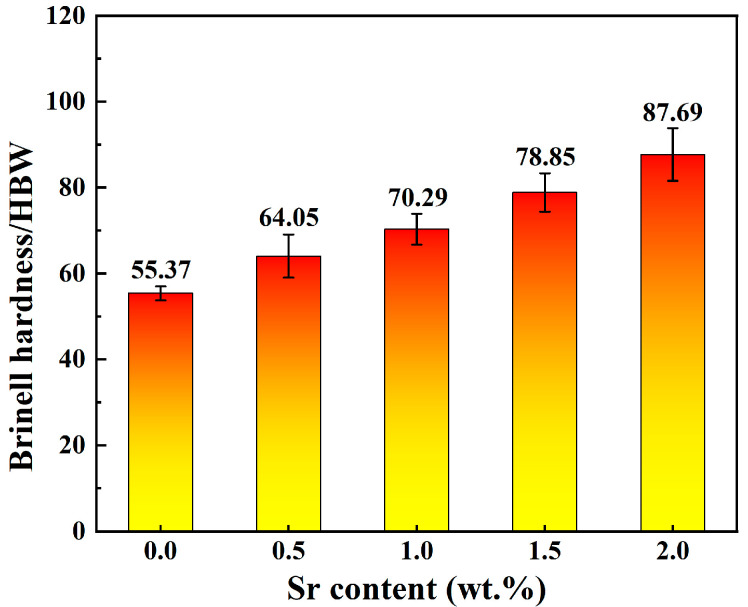
Brinell hardness of Zn-1Fe-*x*Sr alloys.

Figure 5 shows the Brinell hardness of Zn-1Fe-*x*Sr alloys. As shown in Figure 5, the hardness increased significantly with the increase in Sr content. The hardness of Zn-1Fe alloy was 55.37 HBW. The hardness of Zn-1Fe-2Sr alloy was 87.69 HBW, which was 58.4% higher than that of Zn-1Fe alloy. The increase in hardness was mainly due to the grain refinement of η-Zn grains and the SrZn_13_ particles distributed in the η-Zn matrix. As the Sr content increased in Zn-1Fe-*x*Sr alloy, the grain size of η-Zn matrix decreased, and the number of SrZn_13_ particles increased, making the alloy more difficult to deform, and resulting in an increase in the Brinell hardness of the alloy.

#### 3.2.2. Tensile Properties

**Figure 6:**
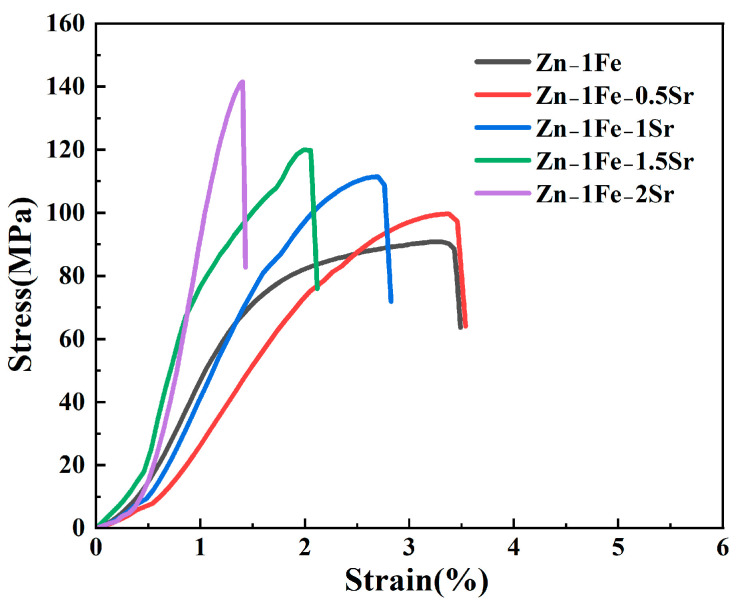
Stress–strain curves of Zn-1Fe-*x*Sr alloys.

**Figure 7:**
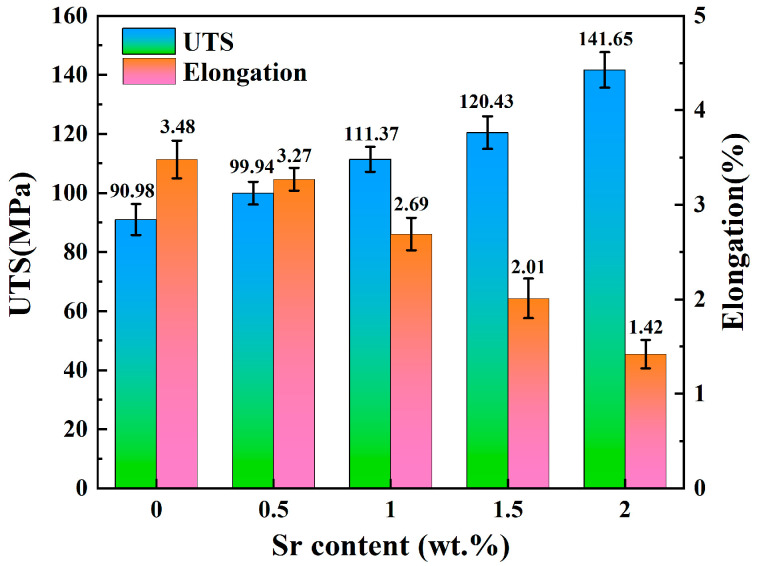
UTS and elongation of Zn-1Fe-*x*Sr alloys.

Figure 6 shows the stress–strain curves of Zn-1Fe-*x*Sr alloys, and the UTS and elongation are exhibited in Figure 7. As illustrated in Figure 6, the tensile stress of Zn-1Fe-*x*Sr alloy increased with the increase in strain, and sharply decreased after reaching the maximum value, indicating that a brittle fracture occurred. As the Sr content increased, the UTS of Zn-1Fe-*x*Sr alloy increased and the elongation decreased. The UTS of Zn-1Fe alloy was 90.98 MPa, and the UTS of Zn-1Fe-2Sr alloy was 141.65 MPa, which was 55.7% higher than the UTS of Zn-1Fe alloy. The increase in UTS could be attributed to two main reasons, fine-grain strengthening and precipitation strengthening. When the Sr element was added, the compositional supercooling zone at the front of the solid–liquid interface became larger. The compositional supercooling zone could promote the nucleation of grains, and the newly formed grains would hinder the growth of other grains [41]. Therefore, after adding the Sr element, the η-Zn grains were refined. The η-Zn grains normally had different orientations, and the adjacent grains with different orientations were separated by grain boundaries. During the plastic deformation of Zn-1Fe-*x*Sr alloy, dislocation motion took place, and the grain boundaries played a role in hindering dislocation motion. The fine-grained alloy had a larger total grain boundary area, and was more difficult to deform, resulting in higher UTS. In addition, the precipitation strengthening was achieved by the formation of FeZn_13_ and SrZn_13_ phases. FeZn_13_ and SrZn_13_ particles were hard obstacles dispersed in the matrix, and impeded the movement of dislocations, leading to an increase in the UTS of Zn-1Fe-*x*Sr alloy. Therefore, with the increase in Sr content, the grain size of Zn-1Fe-*x*Sr alloy decreased, the number of SrZn_13_ particles increased, and the UTS increased.

For the Zn-1Fe-*x*Sr alloy, the Zn matrix was relatively soft, and the FeZn_13_ and SrZn_13_ phases were brittle and hard [42,43]. During the tensile test, the slip of dislocations occurred within a single grain, and dislocations tended to pile up at phase boundaries, i.e., the boundaries between the Zn grains and SrZn_13_ phase or FeZn_13_ phase. The slip of dislocations could be effectively restricted by the phase boundaries, and high stress concentrations could be induced. When the external stress was large enough, high stress concentrations might occur, and tensile cracks started to initiate at the phase boundaries and then propagated [44]. As the Sr content increased, the number of stress concentration points increased, and the elongation decreased.

### 3.3. Degradation Properties

#### 3.3.1. Electrochemical Behavior

**Figure 8:**
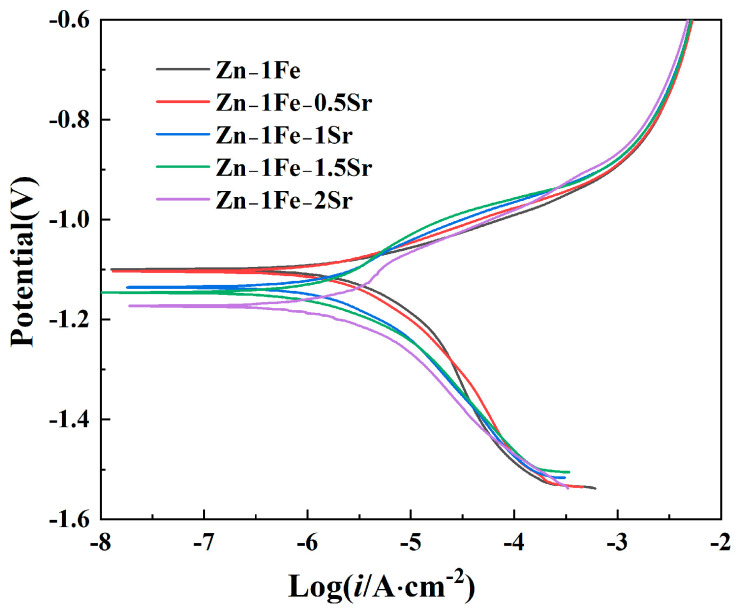
Potentiodynamic polarization curves of Zn-1Fe-*x*Sr alloys.

The degradation behavior of Zn-1Fe-*x*Sr alloys was first investigated by the potentiodynamic polarization curves measured in HBSS, as demonstrated in Figure 8. The Tafel extrapolation method was used to fit the potentiodynamic polarization curves, and the corrosion potential (*E_corr_*) and corrosion current density (*i_corr_*) were obtained, as exhibited in Table 1. Zn-1Fe alloy had a corrosion potential of −1.0997 V, and a corrosion current density of 3.582 μA·cm^−2^. As the Sr content increased, the *E_corr_* of Zn-1Fe-*x*Sr alloy shifted negatively, and the *i_corr_* increased significantly. A more positive *E_corr_* indicated a lower corrosion tendency of the alloy, and a lower *i_corr_* indicated a lower corrosion rate (degradation rate). Therefore, the degradation rate increased significantly with the increase in Sr content. The main reason for the increase in degradation rate was the generation of SrZn_13_ phase. Compared with SrZn_13_ phase and FeZn_13_ phase, the η-Zn matrix had the most negative electrode potential [45,46]. The galvanic corrosion occurred between the Zn grains and the SrZn_13_ and FeZn_13_ phases in the electrochemical test, and the Zn grains were preferentially corroded. As the Sr content increased, the number of SrZn_13_ particles increased, and the degradation rate increased.

**Figure 9:**
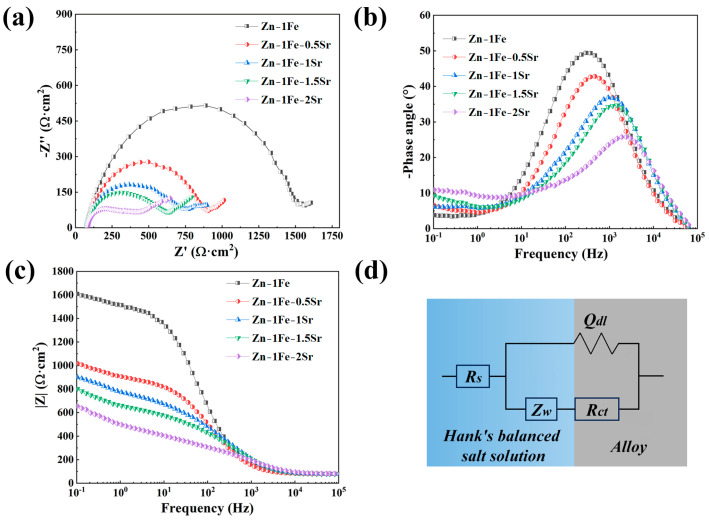
EIS of Zn-1Fe-*x*Sr alloys (**a**) Nyquist plots; (**b**) phase angle plots; (**c**) impedance modulus plots; (**d**) equivalent circuit.

In order to further study the degradation behavior of Zn-1Fe-*x*Sr alloys, the EIS measurements were conducted, and the results are shown in Figure 9. The equivalent circuit which was used to fit the EIS data is shown in Figure 9d. *R_s_* was the resistance of HBSS, and *R_ct_*, *Q_dl_*, and *Z_w_* were the charge transfer resistance, the constant phase element, and the Warburg impedance, respectively. Table 2 shows the detailed fitting results of the EIS data. The *R_ct_* of Zn-1Fe alloy was 1413.1 Ω∙cm^2^, which decreased obviously with the increase in Sr content, indicating that the degradation rate of Zn-1Fe-*x*Sr alloy increased obviously with the increase in Sr content.

#### 3.3.2. Immersion Test

**Figure 10:**
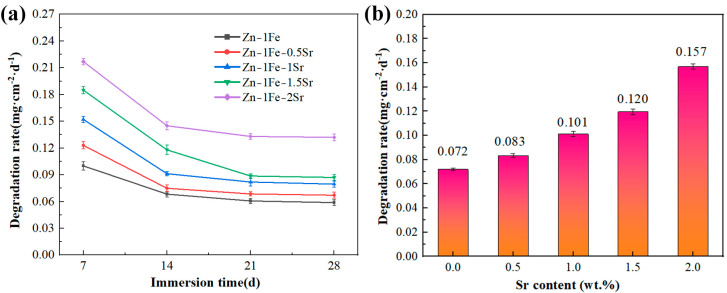
Degradation rate of Zn-1Fe-*x*Sr alloys: (**a**) degradation rate every 7 days; (**b**) the average degradation rate after 28 days of immersion.

Zn-1Fe-*x*Sr alloys were immersed in HBSS and were taken out every 7 days and weighed after the removal of the degradation products. Then, the degradation rate was calculated and shown in Figure 10a. The addition of Sr enhanced the degradation rate of Zn-1Fe-*x*Sr alloy. With the increase in Sr addition, the degradation rate increased obviously, and the Zn-1Fe-2Sr alloy had the highest degradation rate. During the degradation process of Zn-1Fe-*x*Sr alloy, the SrZn_13_ and FeZn_13_ particles served as the cathodes, while the Zn grains served as the anodes and were preferentially degraded. With the increase in Sr addition, the number of SrZn_13_ particles and the degradation rate increased. In addition, the degradation rate of Zn-1Fe-*x*Sr alloy decreased significantly with the increase in immersion time. This may be attributed to the generation of a degradation product film, which was difficult to remove completely and acted as a protective layer, resulting in a decrease in degradation rate [46].

In order to study the long-term degradation performance of Zn-1Fe-*x*Sr alloys, the alloys were immersed in HBSS for 28 days and were taken out and weighed after the degradation products were removed. Then, the degradation rate was calculated and exhibited in Figure 10b. The degradation rate of Zn-1Fe-*x*Sr alloy was improved significantly by the addition of Sr. Zn-1Fe alloy had a degradation rate of 0.072 mg·cm^−2^·d^−1^, and Zn-1Fe-2Sr alloy had a degradation rate of 0.157 mg·cm^−2^·d^−1^. The degradation rate of Zn-1Fe-2Sr increased by 118.1% compared with Zn-1Fe alloy. The results of the immersion tests were in accord with those of the electrochemical tests.

**Figure 11:**
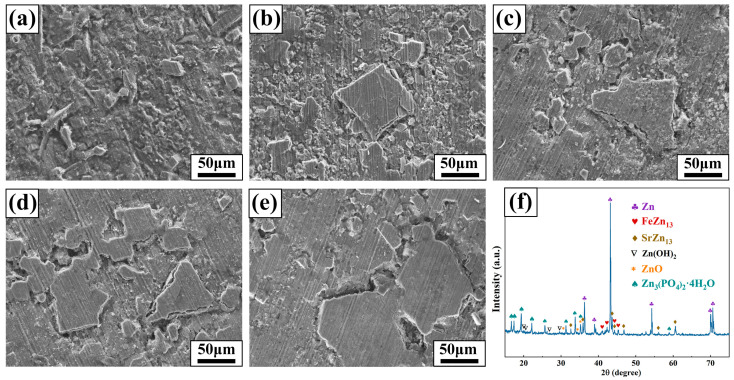
Morphology of samples before removal of degradation products (**a**) Zn-1Fe; (**b**) Zn-1Fe-0.5Sr; (**c**) Zn-1Fe-1Sr; (**d**) Zn-1Fe-1.5Sr; (**e**) Zn-1Fe-2Sr; (**f**) XRD pattern of Zn-1Fe-2Sr alloy before removal of degradation products.

The morphology of Zn-1Fe-*x*Sr alloys after 28 days of immersion in HBSS is displayed in Figure 11. As shown in Figure 11a–e, all samples were covered with degradation products generated during the degradation process. XRD analysis was performed on Zn-1Fe-2Sr alloy to study the composition of degradation products, and the results are displayed in Figure 11f. The diffraction peaks of Zn_3_(PO_4_)_2_·4H_2_O, Zn(OH)_2_, and ZnO were observed, indicating that the degradation products mainly consisted of Zn(OH)_2_, ZnO, and Zn_3_(PO_4_)_2_·4H_2_O.

When the Zn-1Fe-*x*Sr alloy was immersed in HBSS, an oxygen absorption corrosion process occurred, which could be expressed by the following equations [47,48]:

Zn → Zn^2+^ + 2 e^−^ (3)

2 H_2_O + O_2_ + 4 e^−^ → 4 OH^−^ (4)

2 Zn + 2 H_2_O + O_2_ → 2 Zn(OH)_2_ (Overall reaction) (5)

Zn(OH)_2_ → ZnO + H_2_O (6)

The degradation products (corrosion products) Zn(OH)_2_ and ZnO accumulated on the sample surface during the degradation process of the sample, and a layer of degradation products was formed. However, the Cl^−^ ions in HBSS could attack the degradation products such as Zn(OH)_2_, resulting in the decomposition of Zn(OH)_2_ as shown by the following equation [49]:

Zn(OH)_2_ + Cl^−^ → Zn^2+^ + 2 OH^−^ + Cl^−^ (7)

In addition, there were HPO_4_^2−^ anions in HBSS. Zn^2+^ might react with them, leading to the formation of Zn_3_(PO_4_)_2_·4H_2_O. The relevant reaction equation was as follows:

3 Zn^2+^ + 2 HPO_4_^2−^ + 2OH^−^ +2 H_2_O → Zn_3_(PO_4_)_2_·4H_2_O (8)

Some studies have also found the presence of calcium phosphate and zinc (calcium) carbonate salts in degradation products [50]. In this study, no diffraction peaks of other products were observed, possibly due to the low content. These degradation products were formed and accumulated on the sample, and a degradation product film was formed. The degradation product film served as a barrier, hindering the degradation reaction and reducing the degradation rate [47,48,50].

**Figure 12:**
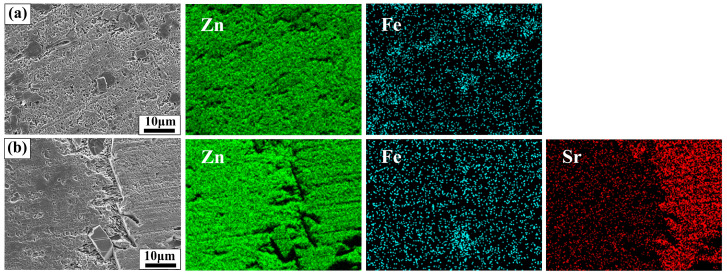
Morphology and EDS maps of Zn-1Fe-*x*Sr alloy after the degradation products were removed (**a**) Zn-1Fe; (**b**) Zn-1Fe-2Sr.

After 28 days of immersion, the SEM images and EDS analysis of Zn-1Fe alloy and Zn-1Fe-2Sr alloy after removal of degradation products are displayed in Figure 12. As shown in Figure 12, the granular FeZn_13_ phase and the blocky SrZn_13_ phase were distributed in the η-Zn matrix. However, the Zn matrix was severely corroded, especially in the area adjacent to the SrZn_13_ and FeZn_13_ phases. Compared with SrZn_13_ phase and FeZn_13_ phase, the Zn matrix had a lower electrode potential, and acted as an anode during galvanic corrosion, resulting in accelerated corrosion of Zn matrix [45,51]. With the increase in Sr addition, the number of SrZn_13_ particles increased, and the degradation rate of the alloy increased.

## 4. Conclusions

Zn-1Fe-*x*Sr alloys were prepared successfully, and the microstructure, tensile properties, hardness, and in vitro degradation performance were studied in this paper. The main conclusions are as follows:

(1) The irregular blocky SrZn_13_ phase was generated in the η-Zn matrix when the Sr element was added. With the increase in Sr addition, the grain size of the Zn-1Fe-*x*Sr alloy decreased, and the amount of SrZn_13_ phase increased;
(2) The UTS and Brinell hardness of Zn-1Fe-*x*Sr alloy were enhanced significantly by the addition of Sr element. With the increase in Sr addition, the UTS and hardness increased. The UTS and hardness of Zn-1Fe-2Sr alloy were 141.65 MPa and 87.69 HBW, which were 55.7% and 58.4% higher than those of Zn-1Fe alloy, respectively;
(3) As the Sr content increased, the corrosion current density of Zn-1Fe-*x*Sr alloy increased, and the charge transfer resistance decreased significantly, indicating that the increased degradation rate. Zn-1Fe-2Sr alloy had a degradation rate of 0.157 mg·cm^−2^·d^−1^, which was 118.1% higher than the degradation rate of Zn-1Fe alloy;
(4) With the increase in immersion time, the degradation rate of Zn-1Fe-*x*Sr alloy decreased significantly. This was mainly due to the generation of a degradation product film on the alloy, which served as a protective layer, leading to a decrease in degradation rate.

## Figures and Tables

**Table 1:** Tafel fitting results based on the potentiodynamic polarization curves of Zn-1Fe-*x*Sr alloys.

Alloy	*E_corr_*/V	*i_corr_*/μA·cm^−2^	Corrosion Rate/mm·Year^−1^
Zn-1Fe	−1.0997	3.582	0.0536
Zn-1Fe-0.5Sr	−1.1038	4.393	0.0658
Zn-1Fe-1Sr	−1.1355	4.832	0.0723
Zn-1Fe-1.5Sr	−1.1461	5.108	0.0764
Zn-1Fe-2Sr	−1.1729	5.596	0.0837

**Table 2:** EIS fitting results.

Samples	*R_s_*	*R_ct_*	*Q_dl_*	
	(Ω·cm^2^)	(Ω·cm^2^)	Y_0_(Ω^−1^·s^n^·cm^−2^)	n
Zn-1Fe	77.93	1413.1	7.0 × 10^−6^	0.800
Zn-1Fe-0.5Sr	76.81	785.4	8.4 × 10^−6^	0.802
Zn-1Fe-1Sr	74.71	634.3	10.9 × 10^−6^	0.725
Zn-1Fe-1.5Sr	76.15	517.1	10.7 × 10^−6^	0.723
Zn-1Fe-2Sr	75.72	354.7	32.5 × 10^−6^	0.603

## Data Availability

The raw data supporting the conclusions of this article will be made available by the authors on request.
